# G-CSF increases calprotectin expression, liver damage and neuroinflammation in a murine model of alcohol-induced ACLF

**DOI:** 10.3389/fcell.2024.1347395

**Published:** 2024-02-14

**Authors:** Martí Ortega-Ribera, Yuan Zhuang, Veronika Brezani, Prashanth Thevkar Nagesh, Radhika S. Joshi, Mrigya Babuta, Yanbo Wang, Gyongyi Szabo

**Affiliations:** ^1^ Department of Medicine, Division of Gastroenterology, Beth Israel Deaconess Medical Center and Harvard Medical School, Boston, MA, United States; ^2^ Broad Institute, Cambridge, MA, United States

**Keywords:** acute-on-chronic liver failure, inflammasome, S100a8/S100a9, neutrophil, BDL, cerebellum, encephalopathy, ethanol

## Abstract

**Background and aims:** Granulocyte colony-stimulating factor (G-CSF) has been proposed as a therapeutic option for patients with ACLF, however clinical outcomes are controversial. We aimed at dissecting the role of G-CSF in an alcohol-induced murine model of ACLF.

**Methods**: ACLF was triggered by a single alcohol binge (5 g/kg) in a bile duct ligation (BDL) liver fibrosis model. A subgroup of mice received two G-CSF (200 μg/kg) or vehicle injections prior to acute decompensation with alcohol. Liver, blood and brain tissues were assessed.

**Results:** Alcohol binge administered to BDL-fibrotic mice resulted in features of ACLF indicated by a significant increase in liver damage and systemic inflammation compared to BDL alone. G-CSF treatment in ACLF mice induced an increase in liver regeneration and neutrophil infiltration in the liver compared to vehicle-treated ACLF mice. Moreover, liver-infiltrating neutrophils in G-CSF-treated mice exhibited an activated phenotype indicated by increased expression of CXC motif chemokine receptor 2, leukotriene B4 receptor 1, and calprotectin. In the liver, G-CSF triggered increased oxidative stress, type I interferon response, extracellular matrix remodeling and inflammasome activation. Circulating IL-1β was also increased after G-CSF treatment. In the cerebellum, G-CSF increased neutrophil infiltration and S100a8/9 expression, induced microglia proliferation and reactive astrocytes, which was accompanied by oxidative stress, and inflammasome activation compared to vehicle-treated ACLF mice.

**Conclusion:** In our novel ACLF model triggered by alcohol binge that mimics ACLF pathophysiology, neutrophil infiltration and S100a8/9 expression in the liver and brain indicate increased tissue damage, accompanied by oxidative stress and inflammasome activation after G-CSF treatment.

## Introduction

Acute-on-chronic liver failure (ACLF) is a dreadful syndrome occurring in patients with chronic liver disease who undergo a sudden hepatic decompensation. ACLF is characterized by systemic inflammation, multi-organ damage frequently involving encephalopathy, and high short-term mortality (Arroyo et al., 2020; European Association for the Study of the Liver, 2023). Currently, there is no effective treatment for patients with ACLF besides extracorporeal support systems and organ transplantation. However, liver transplantation may not be an option in the majority of these patients who suffer from alcohol misuse, the primary precipitating factor in ACLF, for requiring a six-month period of abstinence before being eligible for a liver transplant, or due to organ shortage (Gustot and Jalan, 2019). Consequently, there is an urgent and unmet need for novel therapeutic options in ACLF.

In the last decade, granulocyte colony-stimulating factor (G-CSF) has emerged as a potential therapeutic option for patients with ACLF (Butt and Jalan, 2023). G-CSF is a cytokine that stimulates the production and release of granulocytes, particularly neutrophils from the bone marrow (Mehta and Corey, 2021). In the context of ACLF, G-CSF is being explored for its ability to enhance liver regeneration (proliferation of hepatocytes and oval stem cells in the liver) and improve neutrophil function, as ACLF often involves severe liver dysfunction, impaired regenerative response, and immune paralysis (Piscaglia et al., 2007; Theyab et al., 2021).

Clinical studies and trials using G-CSF showed diverse outcomes on the survival of patients with ACLF. Studies from Asia showed improved survival, MELD and SOFA scores, and prevention of extrahepatic complications including sepsis, hepatorenal syndrome and encephalopathy events in patients with ACLF that received G-CSF (Garg et al., 2012; Duan et al., 2013; Saha et al., 2017; Tong et al., 2022). However, these beneficial effects have not been replicated in studies using G-CSF in Europe (Newsome et al., 2018; Engelmann et al., 2021) or meta-analysis (Di Martino et al., 2023; Martin-Mateos et al., 2023).

At the molecular level, G-CSF has demonstrated the ability to shift monocytes towards a pro-resolving phenotype (M2-like) in HBV-ACLF patients (Tong et al., 2022), to mobilize CD34^+^ hematopoietic stem cells giving rise to new hepatocytes (Li et al., 2010; Garg et al., 2012) and to exacerbate inflammatory response further inducing senescence and cell death in an LPS-induced murine model of ACLF (Engelmann et al., 2022). Nevertheless, the effect of G-CSF in the context of alcohol, mimicking the most prevalent etiology triggering this syndrome, remains unexplored.

Given the controversial results observed in ACLF patients treated with G-CSF and the lack of a comprehensive mechanistic understanding of G-CSF in relevant alcohol-related models of ACLF, this manuscript aims to dissect the role of G-CSF in a novel murine model of alcohol-induced ACLF.

## Results

### A novel model of alcohol-induced ACLF triggers liver damage and inflammation

**FIGURE 1:**
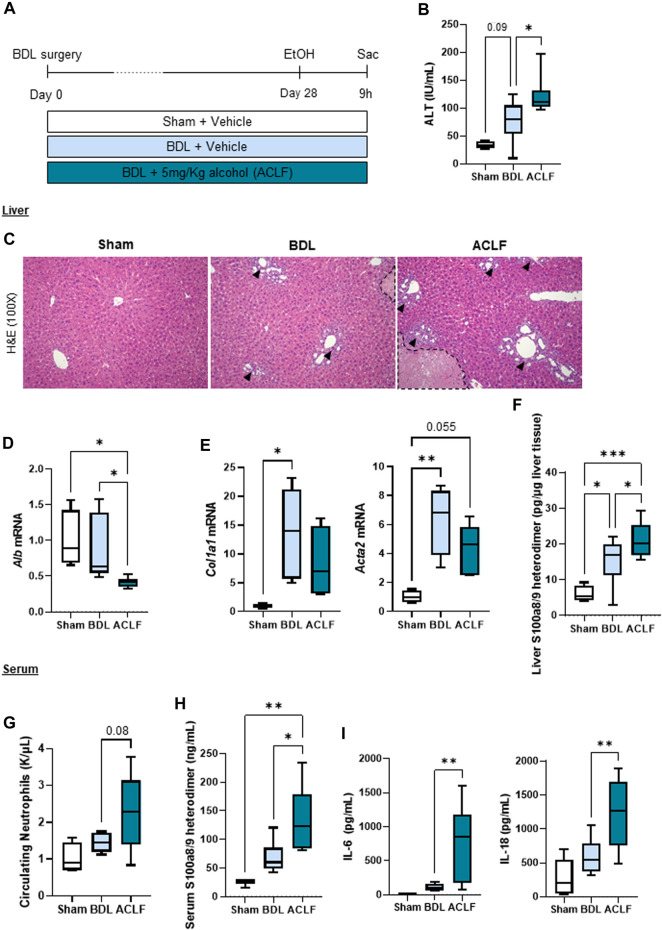
A novel model of alcohol-induced ACLF triggers liver damage and inflammation. **(A)** Schematics for the development of our murine model of ACLF and control groups. **(B)** ALT serum levels. **(C)** Hematoxylin and eosin staining in liver sections. Ductular reaction in portal triads are highlighted by arrowheads and necrotic areas are identified within the dashed lines. **(D)** Hepatic mRNA albumin expression. **(E)** Hepatic mRNA expression of fibrosis markers including *Col1a1* and *Acta2*. **(F)** Hepatic expression of calprotectin in liver lysates. **(G)** Circulating neutrophils in blood. **(H)** Calprotectin levels in serum. **(I)** IL-6 and IL-18 levels in serum. Data are expressed as mean ± standard deviation (*n* = 4/6 per group). Statistical significance was determined using one-way ANOVA * *p* < 0.05, **p < 0.005, ***p < 0.0005.

In order to induce chronic liver disease, mice underwent bile duct ligation surgery for 28 days. In a subgroup of mice, a single 5 g/kg alcohol binge was given to induce ACLF (Figure 1A). ACLF mice showed increased ALT levels in the circulation (Figure 1B), altered liver histology showing ductular reaction in portal triads (arrowheads) and necrotic areas (dashed lines) (Figure 1C), decreased hepatic albumin mRNA expression (Figure 1D), increased expression of fibrosis markers (Figure 1E), and increased expression of S100 calcium-binding protein A8 (S100a8/9 heterodimer), also known as calprotectin (Figure 1F), in the liver. ACLF mice also showed increased neutrophil count in the blood (Figure 1G), increased circulating levels of calprotectin (Figure 1H), and pro-inflammatory cytokines including interleukin (IL) 6 and IL-18 (Figure 1I) indicating systemic inflammation.

### G-CSF increases circulating neutrophils and liver regeneration in alcohol-induced ACLF

**FIGURE 2:**
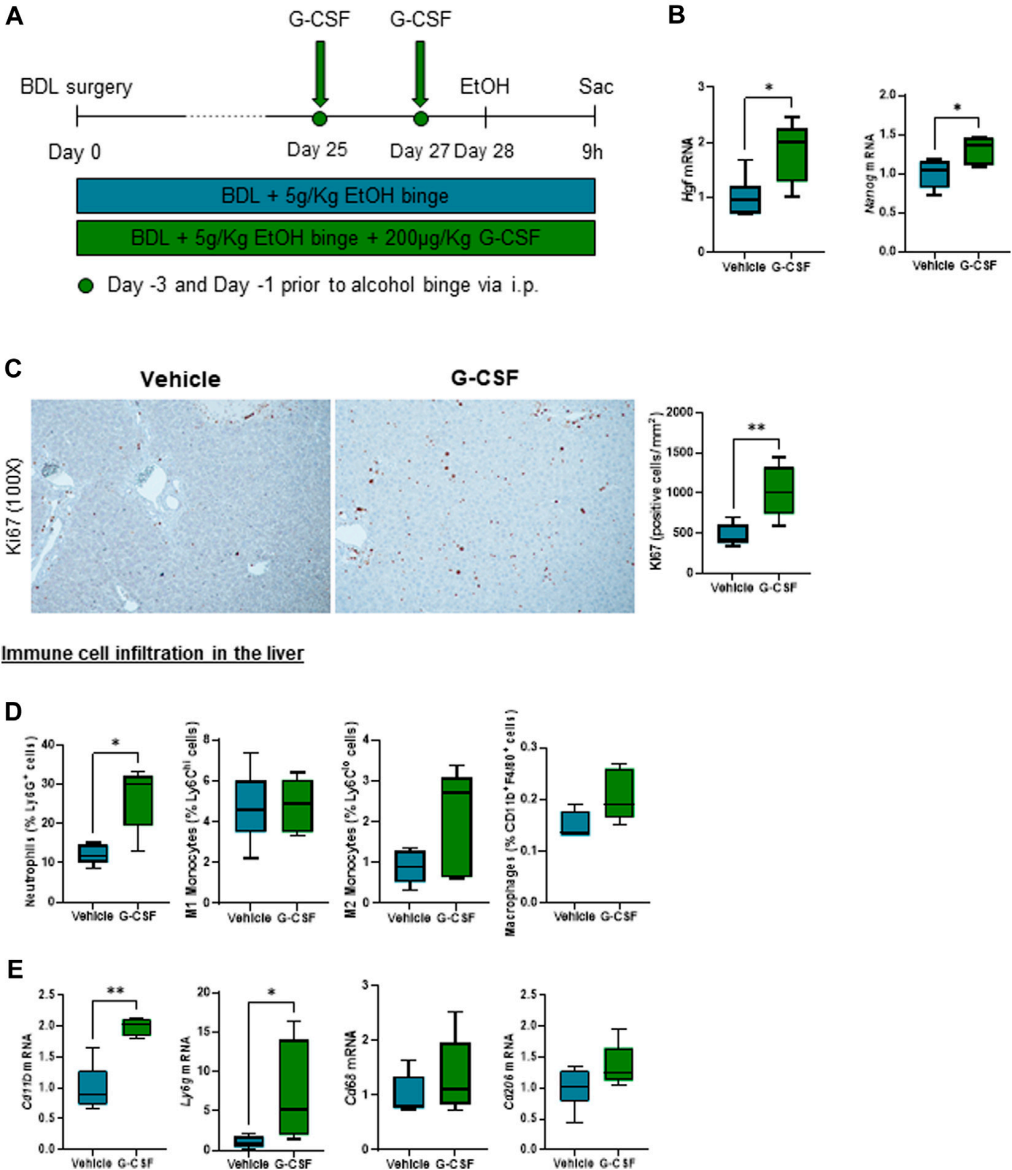
G-CSF increases neutrophil infiltration and liver regeneration in alcohol-induced ACLF. **(A)** Schematics for G-CSF treatment in ACLF mice. **(B)** Hepatic mRNA expression of regeneration markers including *Hgf* and *Nanog*. **(C)** Representative images and quantification of Ki67 immunohistochemistry in the liver measured as the number of positive cells/mm^2^. **(D)** Neutrophil, monocyte and macrophage count form leukocytes isolated from livers in ACLF mice treated with G-CSF or vehicle analyzed by flow cytometry. **(E)** Hepatic mRNA expression of leukocyte markers including *Cd11b*, *Ly6g*, *Cd68* and *Cd206*. Data are expressed as mean ± standard deviation (*n* = 5/6 per group). Scale bar for 100 X: 100 μm. Statistical significance was determined using one-way ANOVA * *p* < 0.05, **p < 0.005.

G-CSF was administered to a subgroup of ACLF mice by intraperitoneal injection on day 25 and 27 after BDL (Figure 2A). G-CSF treatment increased gene expression of regenerative markers in the liver including hepatocyte growth factor (*Hgf*) and nanog homeobox (*Nanog*) (Figure 2B), and expression of proliferation Ki-67 assessed by immunohistochemistry staining in the liver (Figure 2C). Moreover, flow cytometry analysis of liver-infiltrated cells revealed an increase in neutrophils in the liver without significant changes in the monocyte/macrophage populations (Figure 2D). At the mRNA level, hepatic expression of the pan-leukocyte marker, *Cd11b,* and the neutrophil marker, *Ly6g,* were significantly increased after G-CSF treatment without changes in the pro-inflammatory/-resolutive macrophage markers, *Cd68* and *Cd206* (Figure 2E). This suggested that G-CSF treatment in our ACLF model increases liver regeneration and neutrophil recruitment in the liver.

### G-CSF induces neutrophil activation and increased calprotectin expression in the liver

**FIGURE 3:**
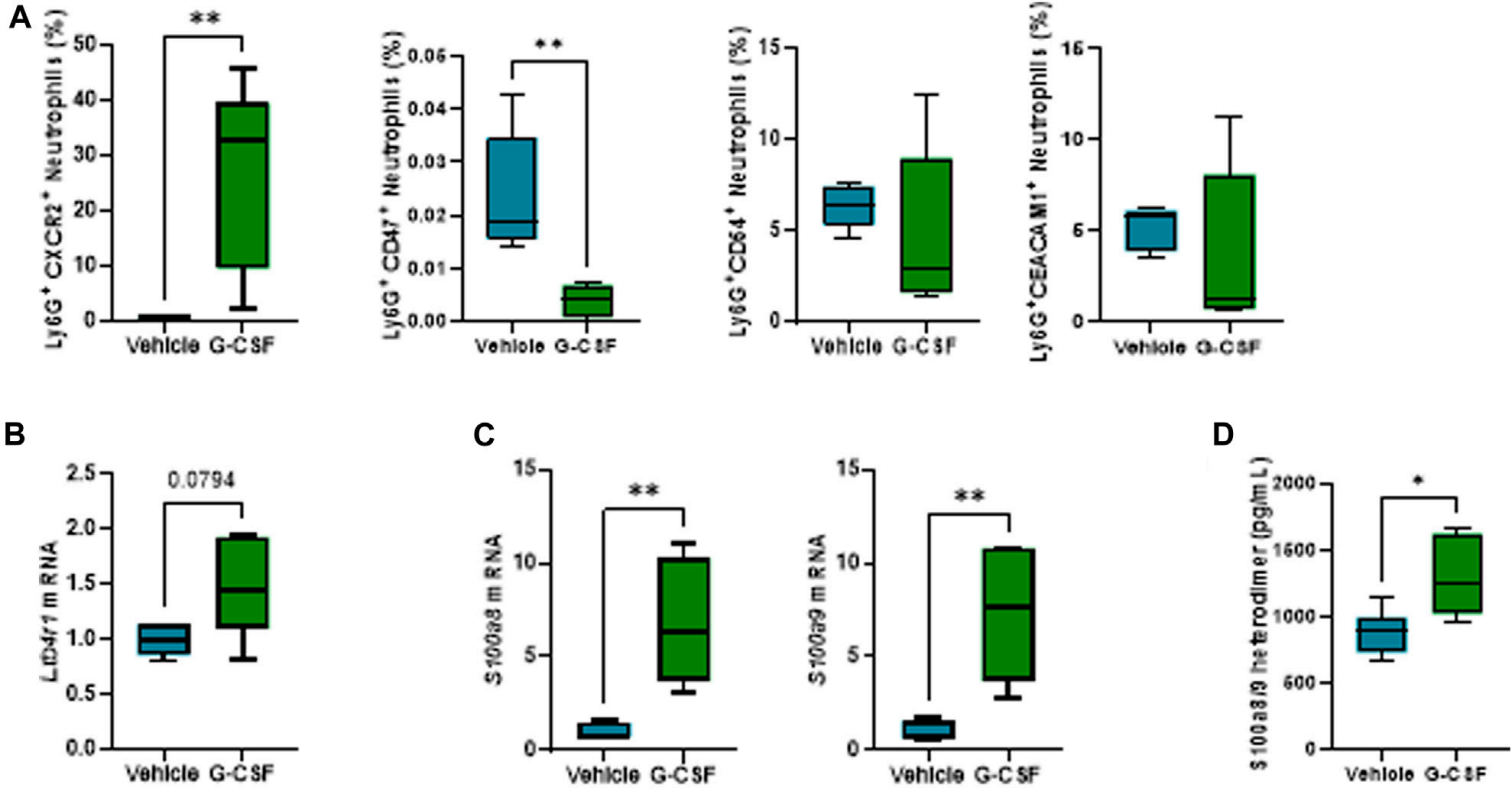
G-CSF induces neutrophil activation and increased calprotectin expression in the liver. **(A)** Neutrophil activation markers analyzed in liver-infiltrated neutrophils by flow cytometry. **(B)** Hepatic mRNA expression of *Ltb4r1*. **(C)** Hepatic mRNA expression of calprotectin components *S100a8* and *S100a9*. **(D)** Calprotectin levels in liver lysates. Data are expressed as mean ± standard deviation (n = 5/6 per group). Statistical significance was determined using one-way ANOVA * *p* < 0.05, **p < 0.005.

In-depth characterization of neutrophil phenotype after G-CSF treatment revealed an increased expression of the chemokine receptor CXC motif chemokine receptor (CXCR) 2 and decreased expression of the “do not eat me” signal CD47 in neutrophils. Moreover, there was no significant change in the neutrophil expression of the adhesion molecules, CD54 or CEA, cell adhesion molecule (CEACAM) 1 after the G-CSF treatment (Figure 3A). In addition, hepatic expression of the neutrophil activation marker, leukotriene 4 receptor (*Ltb4r1*), was increased after G-CSF treatment (Figure 3B). Expression of calprotectin, both at the mRNA (Figure 3C) and protein (Figure 3D) level, was greatly and significantly increased in the liver of ACLF mice that received G-CSF compared to the vehicle-treated group. This data suggests that upon G-CSF treatment, activated neutrophils are recruited to the injured liver in ACLF.

### Oxidative stress, type I interferon response, extracellular matrix remodeling and inflammasome activation are increased in the liver of G-CSF-treated mice

Calprotectin is known to account for around 50% of the cytosolic proteins in neutrophils, serving as an activation and NETosis marker for neutrophils (Pruenster et al., 2016). Calprotectin has been shown to induce cytokine release and inflammasome activation via ROS production in autoimmune inflammatory diseases (Simard et al., 2013), myocardial infarction (Sreejit et al., 2020), and acute kidney injury (Tan et al., 2017); nevertheless their role in ACLF has been unknown to date.

**FIGURE 4:**
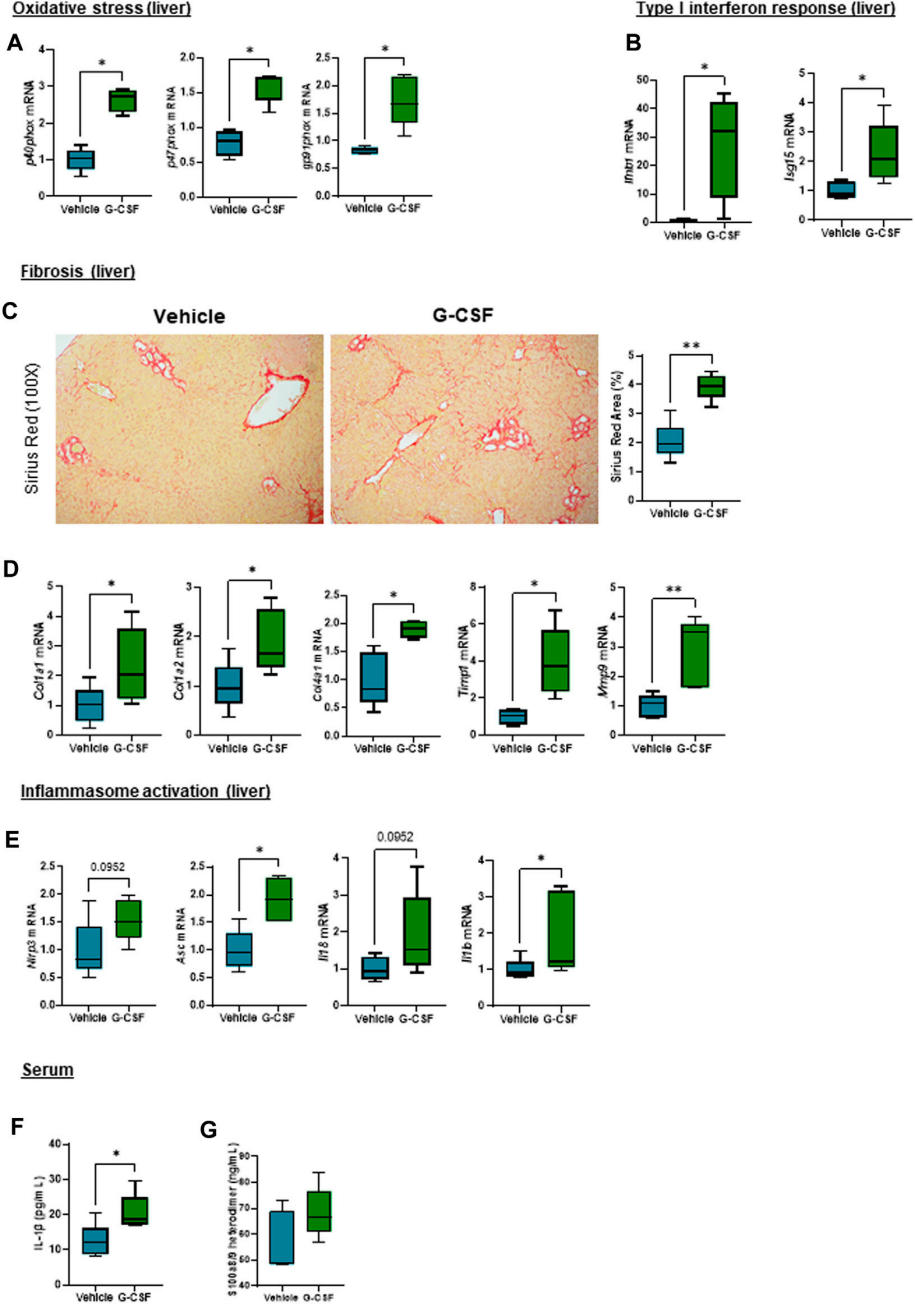
Oxidative stress, type I interferon response, extracellular matrix remodeling and inflammasome activation are increased after G-CSF treatment in the liver. **(A)** Hepatic mRNA expression of oxidative stress markers including *p40phox*, *p47phox* and *gp91phox*. **(B)** Hepatic mRNA expression of type I interferon response markers including *Ifnb1* and *Isg15*. **(C)** Sirius Red staining in liver sections. **(D)** Hepatic mRNA expression of fibrosis/ECM remodeling markers including *Col1a1*, *Col1a2*, *Col4a1*, *Timp1* and *Mmp9*. **(E)** Hepatic mRNA expression of inflammasome markers including *Nlrp3*, *Asc*, *Il18* and *Il1b*. **(F)** IL-1β levels in serum. **(G)** Calprotectin levels in serum. Data are expressed as mean ± standard deviation (*n* = 5/6 per group). Statistical significance was determined using one-way ANOVA * *p* < 0.05, **p < 0.005.

Given the observation that G-CSF induces an increase in the expression of calprotectin, we next explored liver damage pathways in our ACLF model. We observed an activation of oxidative stress pathways as shown by increased mRNA expression of the NADPH oxidase subunits *p40phox*, *p47phox* and *gp91phox* (Figure 4A) and genes involved in type I interferon response including interferon beta (*Ifnβ*) and interferon-stimulated gene 15 (*Isg15*) in ACLF mice that received G-CSF compared to saline treated ACLF controls (Figure 4B). Moreover, liver fibrosis markers including Sirius Red staining (Figure 4C) and mRNA expression of *collagen 1α1, 1α2* and *4α1* and the extracellular matrix remodeling enzymes *Timp1* and *Mmp9* were increased in G-CSF treated ACLF mice (Figure 4D). Additionally, inflammasome activation genes including *Nlrp3, Asc, Il18* and *Il1β* were upregulated in the liver of ACLF mice treated with G-CSF (Figure 4E). Increased calprotectin and inflammasome activation in G-GSF treated ACLF mice was also indicated by significantly higher circulating levels of IL-1β (Figure 4F) and calprotectin (Figure 4G) in the serum of ACLF mice treated with G-CSF compared to vehicle.

### G-CSF induces neutrophil infiltration, increases calprotectin expression and activates inflammasome in the cerebellum of ACLF mice

**FIGURE 5:**
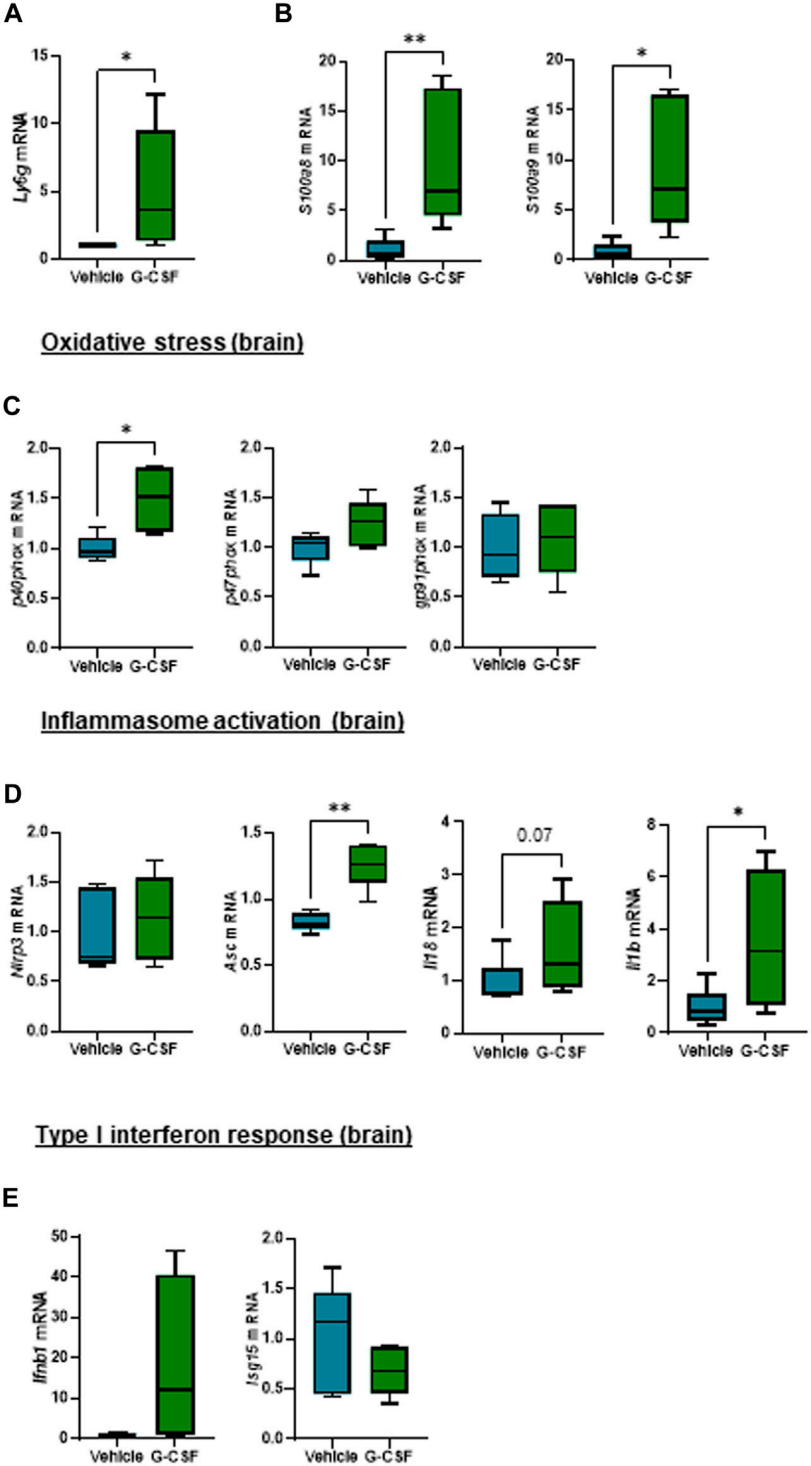
G-CSF induces neutrophil infiltration, increases calprotectin expression and activates inflammasome in the cerebellum of ACLF mice. **(A)** Cerebellum mRNA expression of *Ly6g*. **(B)** Cerebellum mRNA expression of calprotectin components *S100a8* and *S100a9*. **(C)** Cerebellum mRNA expression of oxidative stress markers including *p40phox*, *p47phox* and *gp91phox*. **(D)** Hepatic mRNA expression of inflammasome markers including *Nlrp3*, *Asc*, *Il18* and *Il1b*. **(E)** Hepatic mRNA expression of type I interferon response markers including *Ifnb1* and *Isg15*. Data are expressed as mean ± standard deviation (*n* = 5/6 per group). Statistical significance was determined using one-way ANOVA * *p* < 0.05, **p < 0.005.

Given that hepatic encephalopathy is a common extrahepatic organ dysfunction in ACLF, observation of increased systemic IL-1β prompted us to explore a potential effect of systemic inflammation in the brain of the ACLF mice. The cerebellum was analyzed because it is the most sensitive brain region to ammonia and inflammatory mediators, a known trigger of encephalopathy during ACLF (Parvez and Ohtsuki, 2022). Similarly to findings in the liver, cerebellum from ACLF mice treated with G-CSF exhibited increased neutrophil infiltration and activation compared to ACLF alone as shown by the significant increase in the expression of *Ly6g* (Figure 5A) and the calprotectin components *S100a8 and S100a9* (Figure 5B) respectively. Moreover, G-CSF treatment resulted in increased *p40phox* (Figure 5C), *Asc*, *Il18* and *Il1β* (Figure 5D) mRNA expression in the cerebellum, suggesting the induction of oxidative stress and inflammasome activation. No significant change was observed in the type I interferon response upon G-CSF treatment in the brain (Figure 5E).

This highlights the activation of common molecular and cellular pathways including neutrophil infiltration, calprotectin expression and activation of oxidative stress and inflammasome both in the liver and brain in G-CSF-treated ACLF mice.

### G-CSF triggers microglia proliferation and reactive astrocytes in the cerebellum of ACLF mice

**FIGURE 6:**
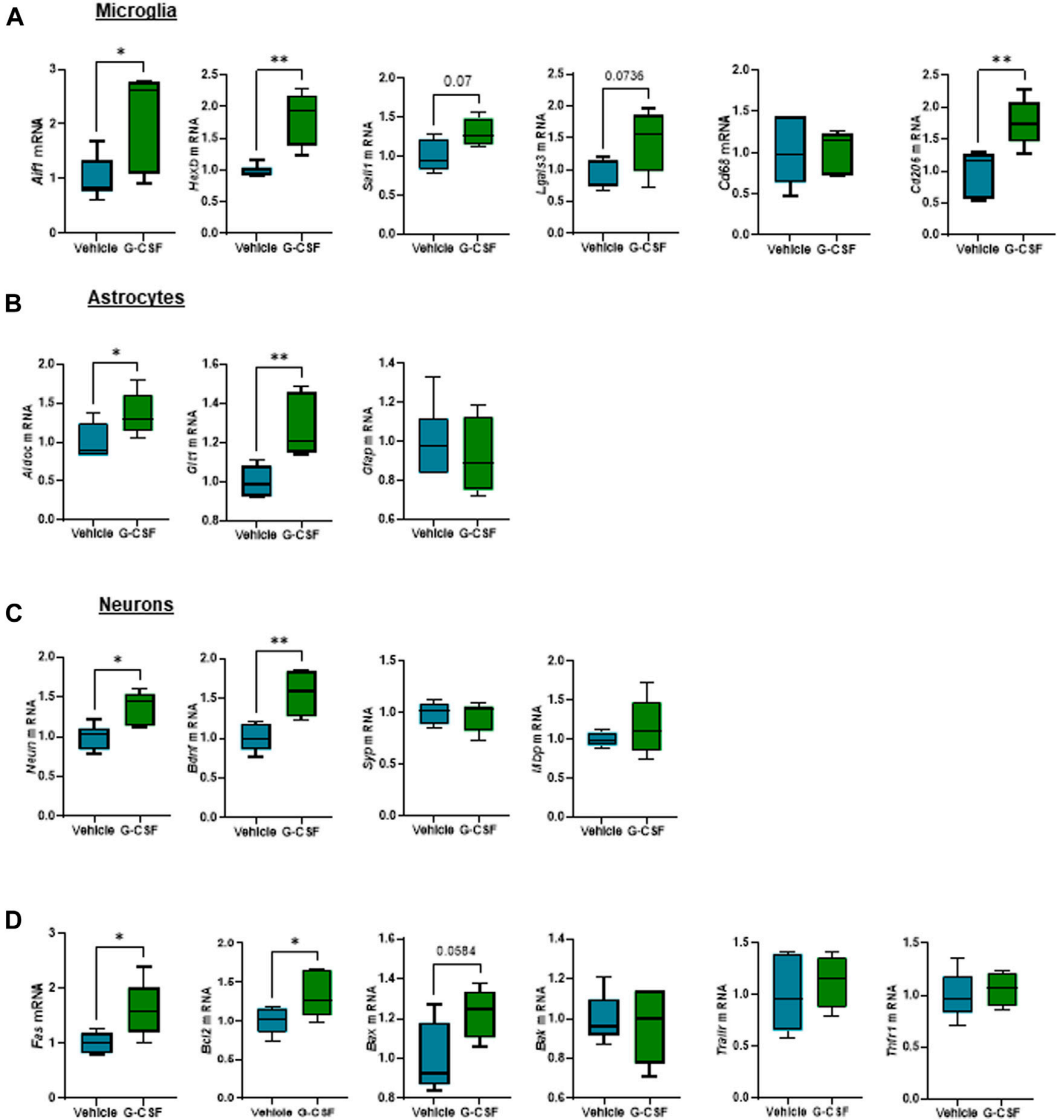
G-CSF triggers microglia proliferation and reactive astrocytes in the cerebellum of ACLF mice. **(A)** Cerebellum mRNA expression of microglia-related markers including *Aif1*, *Hexb, Sall1*, *Lgals3*, *Cd68*, and *Cd206*. **(B)** Cerebellum mRNA expression of astrocyte-related markers including *Aldoc*, *Glt1*, and *Gfap*. **(C)** Cerebellum mRNA expression of neuron-related markers including *Neun*, *Bdnf*, *Syp*, and *Mbp*. **(D)** Cerebellum mRNA expression of cell death markers including *Fas*, *Bcl2*, *Bax*, *Bak*, *Trailr*, *Tnfr1*. Data are expressed as mean ± standard deviation (*n* = 5/6 per group). Statistical significance was determined using one-way ANOVA * *p* < 0.05, **p < 0.005.

We next assessed the response of glial and neuronal cells in the cerebellum to G-CSF treatment. Microglia, resident brain macrophages, become activated and proliferate following brain damage or stimulation (Mander et al., 2006). After ACLF, mice treated with G-CSF showed an increased expression of *Aif1* and microglia-specific markers *Hexb* and *Sall1*, indicating microglia proliferation. We also observed increased expression of *Lgals3* which plays a key role in microglia-mediated neuroinflammation (Ge et al., 2022). There was no change in *Cd68* expression but we found an increase in *Cd206* expression in the G-CSF-treated group (Figure 6A). Furthermore, expression of reactive astrocytes markers *AldoC* and *Glt1*, but not *Gfap,* were increased after G-CSF treatment (Figure 6B). Moreover, we observed increased expression of neuronal markers *Neun* and neurotrophic factor *Bdnf*. There was no significant change in synaptophysin (*Syp*) and myelin binding protein (*Mbp*) after G-CSF treatment when compared to vehicle-treated ACLF mice (Figure 6C). Moreover, the death receptor, *Fas,* and its downstream apoptotic markers, *Bcl2* and *Bax,* were upregulated after G-CSF suggesting an induction of apoptosis in the cerebellum of G-CSF-treated ACLF mice (Figure 6D). No significant change was observed in expression of additional death markers such as *Bak*, *Trailr* or *Tnfr1* (Figure 6D).

## Discussion

In this study, we explored the effect of G-CSF as a therapeutic opportunity utilizing a preclinical model of alcohol-induced ACLF. This novel murine model in cholestatic fibrosis triggered by a single alcohol binge, showed increased liver damage and systemic inflammation compared to BDL-fibrotic mice. Our data indicated that G-CSF treatment in ACLF mice induced an increased regenerative response and neutrophil infiltration in the liver and increased calprotectin expression in both the liver and the brain. In the liver, we found activation of pathways related to oxidative stress, type I interferon response, extracellular matrix remodeling, and inflammasome activation. In the cerebellum, G-CSF triggered increased neutrophil infiltration, oxidative stress, inflammasome induction, and neuroinflammation, overall indicating increased damage after G-CSF treatment in alcohol-induced ACLF.

Given the central role of alcohol as a triggering factor for ACLF (Cullaro et al., 2020), we developed an alcohol-induced murine model of ACLF to further understand the molecular insights of G-CSF treatment in this complex disease. A single 5 g/kg alcohol binge given to BDL-fibrotic mice, triggered ACLF features including elevated ALT, decreased expression of hepatic albumin and increased circulating neutrophils and pro-inflammatory cytokines including IL-6 and IL-18 compared to vehicle -treated fibrotic mice. Importantly, calprotectin levels were significantly increased in both serum and liver lysates from ACLF mice. Consistent with previous reports, treatment with G-CSF increased liver regeneration and neutrophil infiltration in the liver without affecting monocyte/macrophage populations in the liver, proving an efficient G-CSF treatment regime in our murine model. This is likely related to the neutrophil-specific effect of G-CSF on bone marrow precursors and the release of new neutrophils to the circulation (Greenbaum and Link, 2011; Theyab et al., 2021).

Analysis of liver-infiltrating neutrophil phenotype revealed an activated phenotype based on increased expression of the chemokine receptor CXCR2 and reduced expression of the “do not eat me” signal CD47, contributing to neutrophil removal by phagocytes in the liver (Cho et al., 2023). Moreover, increased expression of the neutrophil activation/recruitment markers leukotriene B4 receptor 1 (*Ltb4r1*) and calprotectin indicated neutrophil activation after G-CSF treatment.

Increased calprotectin levels, have been shown in ACLF patients (Matiollo et al., 2022), further validating our ACLF model. Calprotectin (S100a8/9 heterodimer) signaling can be mediated by the activation of TLR4, RAGE or CD33 receptors, inducing NF-kB nuclear translocation, activation of ROS, and translation of chemokine/cytokine genes, which contribute to sustained inflammation (Garcia et al., 2022). Moreover, calprotectin has been shown to induce inflammasome activation in various acute inflammatory diseases (Tan et al., 2017; Sreejit et al., 2020), prompting us to hypothesize the potential role of calprotectin in inflammatory response during G-CSF treatment in ACLF. Our data indicates that G-CSF induces liver damage by activating some of the downstream pathways activated by calprotectin signaling including oxidative stress, type I interferon response, extracellular matrix remodeling and inflammasome activation. Given that hepatic encephalopathy is an important manifestation of the multi-organ ACLF pathophysiology, and that we found increased circulating levels of IL-1β and calprotectin in ACLF mice treated with G-CSF, we next assessed the effect of G-CSF in the cerebellum of ACLF mice. Similar to the liver, we found increased neutrophil infiltration and calprotectin expression after G-CSF treatment, which was accompanied by increased *p40phox* expression and inflammasome activation. NLRP3 is known to play a central role in inflammasome activation and pyroptosis during alcohol-related liver disease (de Carvalho Ribeiro and Szabo, 2022; Brahadeeswaran et al., 2023), nevertheless we cannot exclude contribution of additional pattern-recognition receptors in liver and/or brain of our alcohol-induced ACLF model. Specifically, microglia- and astrocyte-specific markers indicated increased microglia proliferation and reactive astrocytes after G-CSF treatment. Microglia proliferation is part of activation response to damage in the brain (Mander et al., 2006). We also observed increased expression of *Cd206* after G-CSF treatment suggesting microglia M2 polarization and increased expression of neuronal markers and neurotrophic factors, which is in accordance with previous studies (Aschauer-Wallner et al., 2021). Our data also suggests a common activation of damage pathways in response to G-CSF in both the liver and the brain, which we hypothesize to be induced by calprotectin release upon neutrophil infiltration and activation.

Neutrophil activation and calprotectin release upon G-CSF treatment might be beneficial to boost inflammatory response and clear pathogen infection. In AH, G-CSF has been shown to improve survival by increasing the pool of circulating CD34^+^ cells (Singh et al., 2014). However, in an overwhelmed system, such as ACLF, exacerbated inflammatory response with G-CSF might increase organ damage as opposed to promoting tissue repair. Consistent with some recent clinical studies (Newsome et al., 2018; Engelmann et al., 2021; Engelmann et al., 2022), our data indicated that G-CSF does not improve ACLF pathophysiology in a murine model of alcohol-induced ACLF. The deleterious effect of G-CSF has been also shown in non-alcohol models of ACLF (Engelmann et al., 2022) using LPS and GalN as acute triggers for liver damage. Similarly, in the presence of LPS, administration of G-CSF increased systemic inflammation and exacerbated liver damage in a murine model of alcoholic hepatitis (Cho et al., 2022). Nevertheless, the combination of G-CSF treatment and TLR4 inhibition by TAK-242 in a murine model of LPS-induced ACLF mitigated the negative effects of G-CSF treatment, showing an improved phenotype by reducing inflammation and promoting liver regeneration (Engelmann et al., 2022). This observation supports our hypothesis that the combination of G-CSF therapies with others reducing calprotectin signaling might be beneficial during ACLF by promoting liver regeneration but mitigating excessive inflammatory response setting a controlled environment for ACLF recovery. However, it is key to highlight that ACLF is a complex and multifactorial disease, and treatment efficacy, including G-CSF, is dependent on many factors including the underlying cause or trigger of ACLF, the severity of liver damage, timing for G-CSF administration or importantly the presence/absence of infection.

Finally, we observed increased neutrophil infiltration, upregulation of calprotectin and the NLRP3 inflammasome complex in the cerebellum of G-CSF treated ACLF mice. While neutrophils rarely found in the brain, during central nervous system inflammation where the blood-brain-barrier is disrupted, neutrophils are important to contribute to host defense with phagocytosis and can also be found in sterile inflammatory conditions (Shafqat et al., 2023). Neutrophil extracellular traps have been proposed to disrupt the blood-brain barrier (Johnson-Léger et al., 2000). Thus, our observations of increased calprotectin and neutrophil activation likely indicate that G-CSF can augment neuroinflammatory signals in ACLF.

In summary, G-CSF induced liver regeneration and neutrophil infiltration/activation in the liver and brain during alcohol-induced ACLF. We identified calprotectin as a likely central mechanism guiding organ damage in both the liver and the brain during ACLF. Moreover, we describe for the first time the effect of G-CSF in the brain of ACLF mice triggering oxidative stress, inflammasome activation, microglia proliferation/activation and reactive astrocytes. Overall, we conclude that G-CSF does not ameliorate disease pathophysiology in a murine model of alcohol-induced ACLF.

## Materials and methods

### Animals

C57BL/6J mice were purchased from The Jackson Laboratory (Bar Harbor, ME). Mice were housed in a specific pathogen-free mouse facility at Beth Israel Deaconess Medical Center (BIDMC), and all animal handling was performed in compliance with institutional guidelines. Additional procedures were approved by the BIDMC Institutional Animal Care and Use Committee (protocol #010-2019 and #030-2022).

### Bile duct ligation surgery

Bile duct ligation (BDL) surgery, was performed as previously described (Zhuang et al., 2023). Briefly, 10-14- week-old male C57BL/6J mice were anesthetized and placed on an operating pad. Mice were shaved and the skin was disinfected with 70% ethanol. Through an abdominal incision, the common bile duct was identified and ligated. The abdomen and peritoneum were closed with a running silk suture. For sham control mice, the same surgical procedure was performed without the bile duct ligation. Animals were monitored during recovery and treated with buprenorphine (0.1 mg/kg) to avoid pain-induced stress after surgical intervention.

### Alcohol binge administration

Acute administration of ethanol (5 g/kg) by oral gavage was adapted from (Bertola et al., 2013). Briefly, a 47.3% (vol/vol) ethanol solution was prepared from pure alcohol (1000002000, Pharmco) in water. Volume administration to each mouse was calculated as follows: gavage volume (µL) of 47.3% (vol/vol) solution for each mouse = mouse body weight in grams × 15. Control group received water binge.

### Acute-on-chronic liver failure model

In order to develop an alcohol-induced ACLF model, advanced cholestatic liver fibrosis was induced by 28-day BDL surgery followed by a single alcohol binge to trigger acute liver damage (Figure 1A). BDL mice were randomly assigned to receive either alcohol or water as vehicle, those receiving water binge were used as chronic liver disease controls. Consistent with advanced liver disease, 10% of mice developed visible ascites during the last week of BDL and, according to IACUC guidelines, were euthanized and not considered in the study. Nine hours after the alcohol binge, animals were euthanized for sample collection. Specimens were snap frozen in liquid nitrogen and stored at −80°C for molecular analysis or processed in 10% formalin for histology.

### G-CSF treatment

ACLF mice were randomized to receive either 200 µL of saline or G‐CSF (Neupogen; 200 μg/kg body weight) through intraperitoneal injection. G‐CSF injection was performed on days 25 and 27 after BDL surgery.

### ELISA and colorimetric assays

Mice serum or tissue lysate levels of IL-6 (DY406, R&D Systems), IL-18 (DY7625, R&D Systems), S100a8/9 heterodimer (DY8596, R&D Systems) and IL-1β (MHSLB00, R&D Systems) were quantified by using commercially available kits according to the manufacturer’s instructions. ALT levels in serum were measured following manufacturer’s instructions (A525-240, Teco Diagnostics).

### Hemogram

Whole blood was collected in Eppendorf tubes containing 4% sodium citrate and analyzed in Hemavet (M-950HV, Drew Scientific) for quantification of neutrophil population.

### Histology and immunostaining

Liver histology and liver fibrosis were assessed on paraffin-embedded liver sections by Hematoxylin/Eosin and Sirius Red staining respectively. Ki67 was assessed by immunohistochemistry (IHC) on paraffin-embedded liver sections. Briefly, sections were deparaffinated and antigens were exposed by 30min steamer incubation in Tris-EDTA (pH = 9) buffer. After antigen retrieval procedure, endogenous peroxidase activity was inhibited for 10 min with 3% hydrogen peroxide. Sections were blocked for 1 h with normal goat serum and incubated with anti-Ki67 (1:300; 12202S, Cell Signaling Technology) primary antibody O/N at 4°C. HRP-Rabbit (7074S, Cell Signaling Technology) secondary antibody was added. Color development was induced by incubation with a DAB kit (8059S, Cell Signaling Technology) and the sections were counterstained with hematoxylin. Sections were dehydrated and mounted. Positive cell count was performed using QuPath software.

### mRNA isolation and quantitative real-time PCR

Total RNA was extracted and purified with RNeasy^®^ Mini Kit (74106, Qiagen) according to the manufacturer instructions. RNA yield was quantified by Nanodrop ND-1000 spectrophotometer (NanoDrop Technologies, Wilmington, DE, United States). A maximum amount of 1 µg RNA was reverse transcribed using a High-Capacity cDNA Reverse Transcription Kit (1708891BUN, Bio-Rad) in a cDNA Master cycler X50s (Eppendorf), and qPCR was performed in a CFX96 Real-Time PCR System (Bio-Rad), using Sybr Green primers and PCR Master Mix (1725124, Applied Biosystems). RNA expression levels were normalized following the 2^−ΔΔCT^ method, with *gapdh* as housekeeping genes. Detailed primer sequences used for this study can be found in Table 1.

**TABLE 1:** Sequences of real-time PCR primers (Mouse SYBR Green primers).

Gene	Forward sequence (5′-3′)	Reverse sequence (5′-3′)
*Acta2*	GTC​CCA​GAC​ATC​AGG​GAG​TAA	TCG​GAT​ACT​TCA​GCG​TCA​GGA
*Aif1*	CCG​AGG​AGA​CGT​TCA​GCT​AC	GAC​ATC​CAC​CTC​CAA​TCA​GG
*Alb*	CAG​GAT​TGC​AGA​CAG​ATA​GTC	GCTACGGCACAGTGCTTG
*Aldoc*	AGA​AGG​AGT​TGT​CGG​ATA​TTG​CT	TTC​TCC​ACC​CCA​ATT​TGG​CTC
*Asc*	CTG​CTC​AGA​GTA​CAG​CCA​GAA​C	CTG​TCC​TTC​AGT​CAG​CAC​ACT​G
*Bak*	GGA​ATG​CCT​ACG​AAC​TCT​TCA​CC	CAA​ACC​ACG​CTG​GTA​GAC​GTA​C
*Bax*	AGG​ATG​CGT​CCA​CCA​AGA​AGC​T	TCC​GTG​TCC​ACG​TCA​GCA​ATC​A
*Bcl2*	CCT​GTG​GAT​GAC​TGA​GTA​CCT​G	AGC​CAG​GAG​AAA​TCA​AAC​AGA​GG
*Bdnf*	GGC​TGA​CAC​TTT​TGA​GCA​CGT​C	CTC​CAA​AGG​CAC​TTG​ACT​GCT​G
*Cd11b*	TAC​TTC​GGG​CAG​TCT​CTG​AGT​G	ATG​GTT​GCC​TCC​AGT​CTC​AGC​A
*Cd206*	GTT​CAC​CTG​GAG​TGA​TGG​TTC​TC	AGG​ACA​TGC​CAG​GGT​CAC​CTT​T
*Cd68*	TGT​CTG​ATC​TTG​CTA​GGA​CCG	GAG​AGT​AAC​GGC​CTT​TTT​GTG​A
*Col1a1*	GCT​CCT​CTT​AGG​GGC​CAC​T	CCA​CGT​CTC​ACC​ATT​GGG​G
*Col1a2*	TTC​TGT​GGG​TCC​TGC​TGG​GAA​A	TTG​TCA​CCT​CGG​ATG​CCT​TGA​G
*Col4a1*	ATG​GCT​TGC​CTG​GAG​AGA​TAG​G	TGG​TTG​CCC​TTT​GAG​TCC​TGG​A
*Fas*	CTG​CGA​TTC​TCC​TGG​CTG​TGA​A	CAA​CAA​CCA​TAG​GCG​ATT​TCT​GG
*Gapdh*	AGG​TCG​GTG​TGA​ACG​GAT​TTG	TGT​AGA​CCA​TGT​AGT​TGA​GGT​CA
*Gfap*	CAC​CTA​CAG​GAA​ATT​GCT​GGA​GG	CCA​CGA​TGT​TCC​TCT​TGA​GGT​G
*Glt1*	TTC​CAA​GCC​TGG​ATC​ACT​GCT​C	GGA​CGA​ATC​TGG​TCA​CAC​GCT​T
*Gp91phox*	TGG​CGA​TCT​CAG​CAA​AAG​GTG​G	GTA​CTG​TCC​CAC​CTC​CAT​CTT​G
*Hexb*	GCT​GTT​GGT​GAG​AGA​CTC​TGG​A	GAG​GTT​GTG​CAG​CTA​TTC​CAC​G
*Hgf*	GTC​CTG​AAG​GCT​CAG​ACT​TGG​T	CCA​GCC​GTA​AAT​ACT​GCA​AGT​GG
*Ifnb1*	GCC​TTT​GCC​ATC​CAA​GAG​ATG​C	ACA​CTG​TCT​GCT​GGT​GGA​GTT​C
*Il18*	GAC​AGC​CTG​TGT​TCG​AGG​ATA​TG	TGT​TCT​TAC​AGG​AGA​GGG​TAG​AC
*Il1b*	TCT​TTG​AAG​TTG​ACG​GAC​CC	TGA​GTG​ATA​CTG​CCT​GCC​TG
*Isg15*	CAT​CCT​GGT​GAG​GAA​CGA​AAG​G	CTC​AGC​CAG​AAC​TGG​TCT​TCG​T
*Lgals3*	AAC​ACG​AAG​CAG​GAC​AAT​AAC​TGG	GCA​GTA​GGT​GAG​CAT​CGT​TGA​C
*Ltb4r1*	GAC​TTG​GCT​GTG​TTG​CTC​ACT​G	AGC​AGG​ACA​CTG​GCA​TAC​ATG​C
*Ly6g*	GAC​TTC​CTG​CAA​CAC​AAC​TAC​C	ACA​GCA​TTA​CCA​GTG​ATC​TCA​GT
*Mbp*	ATT​CAC​CGA​GGA​GAG​GCT​GGA​A	TGT​GTG​CTT​GGA​GTC​TGT​CAC​C
*Mmp9*	GCT​GAC​TAC​GAT​AAG​GAC​GGC​A	TAG​TGG​TGC​AGG​CAG​AGT​AGG​A
*Nanog*	GAA​CGC​CTC​ATC​AAT​GCC​TGC​A	GAA​TCA​GGG​CTG​CCT​TGA​AGA​G
*Neun*	CAC​CAC​TCT​CTT​GTC​CGT​TTG​C	GGC​TGA​GCA​TAT​CTG​TAA​GCT​GC
*Nlrp3*	TCA​CAA​CTC​GCC​CAA​GGA​GGA​A	AAG​AGA​CCA​CGG​CAG​AAG​CTA​G
*P40phox*	CAA​AGA​CCT​GCT​AGC​GCT​CAT​G	CCA​CAT​CCT​CAT​CTG​ACA​GCA​G
*P47phox*	GCT​GAC​TAC​GAG​AAG​AGT​TCG​G	CCT​CGC​TTT​GTC​TTC​ATC​TGG​C
*S100a8*	CAA​GGA​AAT​CAC​CAT​GCC​CTC​TA	ACC​ATC​GCA​AGG​AAC​TCC​TCG​A
*S100a9*	TGG​TGG​AAG​CAC​AGT​TGG​CAA​C	CAG​CAT​CAT​ACA​CTC​CTC​AAA​GC
*Sall1*	GCT​TGC​ACT​ATC​TGT​GGA​AGA​GC	CTG​GGA​ACT​TGA​CAG​GAT​TGC​C
*Syp*	TTG​GCT​TCG​TGA​AGG​TGC​TGC​A	ACT​CTC​CGT​CTT​GTT​GGC​ACA​C
*Timp1*	TCT​TGG​TTC​CCT​GGC​GTA​CTC​T	GTG​AGT​GTC​ACT​CTC​CAG​TTT​GC
*Tnfr1*	GGG​CAC​CTT​TAC​GGC​TTC​C	GGT​TCT​CCT​TAC​AGC​CAC​ACA

### Flow cytometry

Fifty μL of peripheral blood was collected in Eppendorf tubes containing 0.5M EDTA to prevent coagulation. Blood samples were incubated with Fc block for 10 m on ice (TruStain FcX™, Biolegend, Cat# 101320) followed by a cocktail of antibodies (1:100 dilution) for 30 min on ice; APC/Cyanine7 anti-mouse CD45 Antibody (Biolegend, 103116, clone 30-F11), Brilliant Violet^®^ 711 Anti-CD11b Rat Monoclonal Antibody (Biolegend, Cat#101242, clone: M1/70), Alexa Fluor^®^ 700Anti-Ly-6C Rat Monoclonal Antibody (Biolegend, Cat#128024, clone: HK1.4), PerCP/Cyanine5.5 anti-mouse CXCR2 Antibody (Biolegend, Cat#149307), PE anti-mouse CD47 Antibody (Biolegend, Cat#127507), Alexa Fluor^®^ 594 anti-mouse CEACAM1a Antibody (Biolegend, Cat#134522), Zombie NIR™ Fixable Viability Kit (Biolegend, Cat#423106), and APC anti-mouse CD54 Antibody (Biolegend, Cat#116119). After 30 min, all samples were incubated with BD Phosflow™ Lyse/Fix Buffer (BD Biosciences, 558049) following manufacturer’s protocol. Following lyse/Fix, cells were washed twice with 1× PBS containing 2% FBS, and resuspended in FACS buffer containing 2% FBS in PBS. Samples were run in Aurora Spectral Flow Cytometer (Cytek) and data analysis was done using Flowjo version 8.8.7 software.

### Statistical analysis

Statistical analyses were performed using the Graphpad Prism 9 statistics software for Windows. Normality of sample distribution was assessed using the Kolmogorov-Smirnov test. For samples fitting a normal distribution, means were compared by the Student’s t-test (2 groups) or ANOVA (>2 groups) followed by the Tukey *post hoc* analysis. Otherwise, means were compared using the non-parametric Kruskal-Wallis test followed by the Mann-Whitney U test. Differences were considered significant at *p* < 0.05. Statistical analyses were performed using GraphPad Prism 8.0.2 software (GraphPad Software, Inc.; San Diego, CA, United States).

## Data Availability

The raw data supporting the conclusions of this article will be made available by the authors, without undue reservation.
